# Molecular recognition of itch-associated neuropeptides by bombesin receptors

**DOI:** 10.1038/s41422-022-00743-6

**Published:** 2022-11-03

**Authors:** Changyao Li, Youwei Xu, Heng Liu, Hongmin Cai, Yi Jiang, H. Eric Xu, Wanchao Yin

**Affiliations:** 1 The CAS Key Laboratory of Receptor Research, Shanghai Institute of Materia Medica, Chinese Academy of Sciences, Shanghai, China; 2 University of Chinese Academy of Sciences, Beijing, China; 3 Lingang Laboratory, Shanghai, China; 4 School of Life Science and Technology, ShanghaiTech University, Shanghai, China; 5 Zhongshan Institute for Drug Discovery, Shanghai Institute of Materia Medica, Chinese Academy of Sciences, Zhongshan, Guangdong China

**Keywords:** Cryoelectron microscopy, Extracellular signalling molecules

Dear Editor,

Itch, especially persistent itch (or chronic pruritus) in patients with allergic skin diseases and/or neuropathic problems, significantly affects sleep, mood and individual health. Extensive efforts have been made to develop novel therapeutic strategies to fight itch. Unfortunately, there is no FDA-approved treatment for chronic pruritus despite great efforts in antipruritic research in the past few decades.^1^

Bombesin (Bn) is an amphibian tetradecapeptide found in the skin of *European Bombina*. Two mammalian Bn-like peptides have been characterized, neuromedin B (NMB) and gastrin-releasing peptide (GRP), which are the endogenous ligands for neuromedin B receptor (NMBR or BB_1_ receptor) and gastrin-releasing peptide receptor (GRPR or BB_2_ receptor), respectively.^2^ In addition, there is an orphan bombesin receptor, BRS3 or BB_3_R, which shares sequence and structural similarity with NMBR and GRPR but is not activated by NMB or GRP (Supplementary information, Fig. S1).^2^ Both NMB and GRP are involved in a variety of physiological and pathological processes, including itch induced by histaminergic or nonhistaminergic pruritogens^1,3–7^ (Fig. 1a). As itch-associated neuropeptide receptors in the spinal cord, both NMBR and GRPR are at the core of itch transmission and play a pivotal role in itch biology;^3^ accordingly, they are attractive targets for antipruritic intervention.

**Fig. 1:**
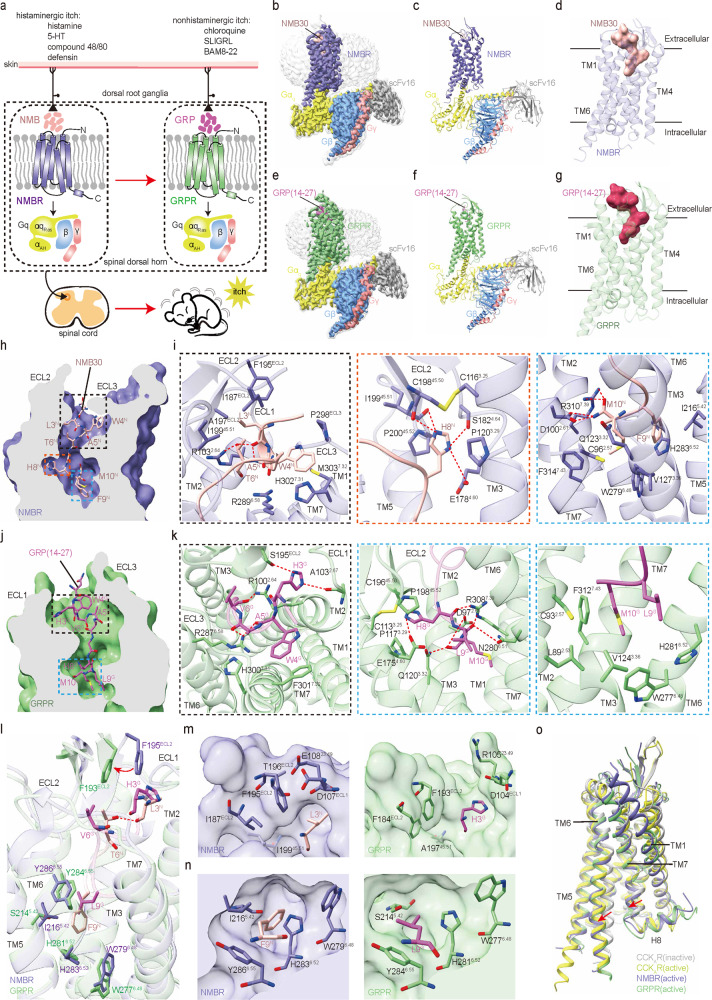
Cryo-EM structures of the NMB30–NMBR–G_q_ and GRP(14–27)–GRPR–G_q_ complexes and analyses of the binding and selectivity for two itch-associated neuropeptides. **a** A schematic illustration of the chemical itch pathways, showing the pivotal roles of the two itch-associated neuropeptides (NMB and GRP) and receptors (NMBR and GRPR) in itch transmission. **b**–**d** Cryo-EM density map (**b**), model (**c**) of the NMB30**–**NMBR**–**G_q_ complex, and the dumbbell-shaped NMB30 in NMBR (**d**). NMB30 is shown in coral; the receptor NMBR is displayed in slate blue. **e**–**g** Cryo-EM density map (**e**), model (**f**) of the GRP(14–27)–GRPR**–**G_q_ complex, and the dumbbell-shaped GRP(14–27) in GRPR (**g**). GRP(14–27) is shown in orchid; the receptor GRPR is displayed in green. The heterotrimeric G_q_ proteins in both structures are colored by subunit: Gα_q_, yellow; Gβ, cornflower blue; Gγ, deep salmon; scFv16, gray. **h**–**k** Cross-section views of the NMB30-binding pocket in NMBR (**h**), and the GRP(14–27)-binding pocket in GRPR (**j**). Detailed interactions of NMB30 with NMBR (**i**), and GRP(14–27) with GRPR (**k**). Key receptor ECLs and TM helixes are annotated, while hydrogen bonds are depicted as red dashed lines. **l** Detailed interactions between L3^N^ and F9^N^ with residues in NMBR and H3^G^ and L9^G^ with residues in GRPR. **m** A comparison of detailed interactions of L3^N^ and H3^G^ and corresponding residues in NMBR and GRPR. **n** A comparison of detailed interactions of F9^N^ and L9^G^ and corresponding residues in NMBR and GRPR. For **l**–**n**, the related residues in the ligands and receptors are shown in stick representation. **o** Structural superposition of two active bombesin receptors, active CCK_A_R (PDB 7EZM) and inactive CCK_A_R (PDB 7F8U). The movement directions of TM6 and TM7 in bombesin receptors relative to inactive CCK_A_R are indicated by red arrows. CCK_A_R, cholecystokinin A receptor. Inactive CCK_A_R, active CCK_A_R, NMBR, and GRPR are colored gray, yellow, slate blue and green, respectively.

To explore the mechanism of peptide recognition by itch receptors, we assembled agonist–bombesin receptor–G_q_ complexes for cryo-electron microscopy (cryo-EM) studies (Supplementary information, Fig. S2) using approaches of BRIL fusion, NanoBiT tethering, G_q_ engineering, and antibody scFv16, which had previously been used successfully in solving several GPCR/G-protein complexes.^8,9^ The cryo-EM structures of NMBR and GRPR, both complexed with the engineered G_q_ and selective peptide agonists NMB30 and GRP(14–27), were solved at global resolutions of 3.15 Å and 3.30 Å, respectively (Fig. 1b–g; Supplementary information, Figs. S3–S5 and Table S1).

In both structures, unambiguous electron densities were observed for the C-terminal 9 residues of both bombesin agonists, which are close to the minimal 10-residue peptide required for full potency.^10^ Compared to the shallow, solvent-exposed pockets in the other class of itch receptors, MRGPRX2/4,^11,12^ both bombesin receptors exhibit classically deep pockets that are commonly found in neuropeptide receptors (Supplementary information, Fig. S6). Each bombesin agonist has a dumbbell-shaped overall conformation and adopts a binding pose perpendicular to the membrane plane (Fig. 1d, g), with its C-terminus inserted deep into the transmembrane domain (TMD) bundle and its N-terminus (referring throughout the paper to the resolved N-terminal amino acids) pointing to the extracellular surface, partially explaining the conserved activation mechanism between these two bombesin receptors. The ligand-recognition region of both receptors can be divided into two major parts: (1) the extracellular loops around the N-terminal dumbbell end and (2) the bottom of the TMD pocket burying the C-terminal dumbbell end (Fig. 1h–k). All positions of bombesin peptides are numbered from the amino terminus of NMB or NMC for clarity (Supplementary information, Table S2).

In the NMBR structure, the N-terminal dumbbell end comprises NLWAT (2–6)^N^ (the superscript N refers to NMB30) residues, forming extensive hydrophobic interactions with residues in the binding pocket (Fig. 1i). In addition, R103^2.64^ forms a close hydrogen bond with T6^N^ and plays a vital role in NMBR activation, which is consistent with our mutagenesis analysis showing that R103^2.64^A decreased the potency of NMB30 to activate NMBR (Supplementary information, Fig. S7a and Table S3). At the bottom of the binding pocket is the C-terminal dumbbell end formed by the HFM motif (Fig. 1i). H8^N^ is well sandwiched by P120^3.29^ and P200^45.52^, which is further stabilized by the conserved disulfide bond between C116^3.25^ and C198^45.50^. H8^N^ also forms hydrogen bonds with E178^4.60^, S182^4.64^, C198^45.50^ and I199^45.51^ (Fig. 1i). Alanine mutations of P120^3.29^, E178^4.60^, and P200^45.52^ had a great influence on receptor activation, especially the P200^45.52^A and P200^45.52^S mutations, suggesting the key role of ECL2 in peptide recognition (Supplementary information, Fig. S7b, c and Table S3). F9^N^ forms a stabilizing π–π packing with H283^6.52^, while F9^N^ and M10^N^ fit into a hydrophobic crevice formed by the residues C96^2.57^, V127^3.36^, W279^6.48^, H283^6.52^, and F314^7.43^ (Fig. 1i). The hydrophobic network here is essential for NMBR activation, since replacing V127^3.36^ with alanine entirely abolished the activity of NMB30 (Supplementary information, Fig. S7c and Table S3). The C-terminal amide forms a hydrogen bond with D100^2.61^, and the C-terminal carbonyl group forms an additional hydrogen bond with R310^7.39^ (Fig. 1i). Alanine replacements of D100^2.61^ and R310^7.39^ displayed a 10- to 50-fold reduction in NMB30 potency (Supplementary information, Fig. S7a, e and Table S3). It is noteworthy that the remarkable conformational shift of the C-terminal amidated end plays a pivotal role in NMB30 potency, since our mutation of Q123^3.32^ to alanine was associated with only a ~30-fold reduction in NMB30 potency, while the mutation of Q123^3.32^ to arginine with a bulky side chain to clash with the C-terminal amidated end completely abolished the ability of NMB30 to stimulate NMBR (Supplementary information, Fig. S7c and Table S3). Together, these detailed structural analyses provide important information to better understand the mechanism of NMB30 recognition by NMBR.

In the GRPR structure, the N-terminus adopts a more extended conformation. The side chains of R100^2.64^ and R287^6.58^ form three hydrogen bonds with the main-chain carbonyl groups of W4^G^, A5^G^, and V6^G^ and drag the N-terminus of GRP(14–27) towards ECL1 and ECL2, where H3^G^ forms a hydrogen bond with the backbone carbonyl of A103^2.67^ and an additional hydrogen bond with the side chain of S195^ECL2^ (Fig. 1k). In addition, W4^G^ forms aromatic packing with H300^7.31^ and F301^7.32^ (Fig. 1k), which further stabilizes the N-terminal bending conformation. At the bottom of the binding pocket is the C-terminal dumbbell end formed by the HLM motif of GRP(14–27) (Fig. 1j, k). H8^G^ is also sandwiched by P117^3.29^ and P198^45.52^, which are further stabilized by the conserved disulfide bond between C113^3.25^ and C196^45.50^ and the hydrogen bond between H8^G^ and E175^4.60^ from TM4 (Fig. 1k). Additionally, R308^7.39^ forms two hydrogen bonds with H8^G^ and L9^G^, while N280^6.51^ forms a hydrogen bond with the backbone carbonyl of L9^G^, and M10^G^ forms two hydrogen bonds with D97^2.61^ and Q120^3.32^ (Fig. 1k). As in the NMBR structure, the side chains of L9^G^ and M10^G^ fit into a hydrophobic crevice formed by the residues L89^2.53^, C93^2.57^, V124^3.36^, W277^6.48^, H281^6.52^, and F312^7.43^ (Fig. 1k). Alanine replacements of V124^3.36^, H281^6.52^, and F312^7.43^ displayed a predominant influence, showing a 3- to 6-fold reduction in GRP(14–27) potency to induce receptor activation (Supplementary information, Fig. S7f–j and Table S3). Together, these results reveal the mechanism of GRP(14–27) recognition and highlight the various peptide-binding modes between the two bombesin receptors.

The above structural insights into the binding pockets encompassing the two peptides reveal that minor amino acid differences between the two receptors reshape their binding modes and allow different peptide preferences, whereas most residues in the binding pockets of NMBR and GRPR are highly conserved (Supplementary information, Figs. S1, S8). NMB30 differs from GRP(14–27) at three residues within the C-terminal decapeptide, and the mutations of L3^N^ and F9^N^ in NMB30 to the equivalent H3^G^ and L9^G^ in GRP(14–27) are known to increase potency and specificity.^10^ Considering the report that NMBR binds to NMB30 with 640-fold higher affinity than GRP(14–27), whereas GRPR binds to GRP(14–27) with 650-fold higher affinity than NMB30,^2^ we performed structural analysis to explore the itch-associated peptide selectivity of NMBR and GRPR. The main differences are located in the ECL2 regions with a deflection of 2.1 Å, as measured between the Cα atoms of the conserved F195^ECL2^ in NMBR and F193^ECL2^ in GRPR (Fig. 1l). The swing of the side chain of F193^ECL2^ in GRPR away from the peptide GRP(14–27) creates sufficient space to fit the bulky side chain of H3^G^ (Fig. 1m), which is reported to be the most important residue for receptor subtype selectivity.^10^ The characteristic conformation of F193^ECL2^ in GRPR is stabilized by the side chain of F184^ECL2^, which is shifted towards A197^45.51^. The corresponding NMBR residues are I187^ECL2^ and I199^45.51^, whose side chains are too rigid to allow similar ECL2 movement as seen in the GRPR structure. NMBR ECL2 is further stabilized by a unique hydrogen bond between T196^ECL2^ and E108^23.49^ (the cognate residue R105^23.49^ in GRPR pointing to the solvent). The locked conformation of F195^ECL2^ in NMBR greatly affects the affinity and activity of bombesin peptides with a His residue at position 3, which is consistent with a detailed study of loss- and gain-of-affinity chimeric GRPR/NMBR.^13^ In contrast, the structural feature of ECL2 in GRPR allows its compatibility with L3 or Q3 in its peptide ligands, which is consistent with systematic studies of 24 peptide ligands at GRPR.^14^ In addition, the selectivity of NMB30 for NMBR is also attributable partly to specific interactions of F9^N^ of NMB30 with the conserved residues W^6.48^, H^6.52^, and Y^6.55^, which form a relatively hydrophobic binding pocket in both receptors (Fig. 1n). This NMBR pocket also contains I216^5.42^, which is directly packed against F9^N^ of NMB30 (Fig. 1n). The corresponding GRPR residue is S214^5.42^ (Fig. 1n), which cannot form similar packing interactions with F9^N^ of NMB30, providing a further explanation of the selectivity of NMBR for NMB30. This is consistent with previous studies with NMBR/GRPR chimeric receptors.^15^ In addition, T6^N^ in NMB30 forms an intramolecular hydrogen bond interaction with L3^N^, which is absent in the structure of GRP(14–27) and has not been previously reported (Fig. 1l). The [V^6^] NMB showed a ~11-fold decrease in binding affinity to NMBR,^10^ suggesting that the conformation of NMB30 assisted by this unique intramolecular hydrogen bond interaction also plays a role in receptor subtype selectivity. Together, these data reveal the determinants of bombesin receptor selectivity between NMB30 and GRP(14–27).

A structural comparison of the NMBR and GRPR complexes to their closely related cholecystokinin A receptor (CCK_A_R) in the active (PDB 7EZM) and inactive states (PDB 7F8U) sheds light on the basis of bombesin receptor activation. Both NMBR and GRPR adopt fully active conformations similar to the active CCK_A_R (Fig. 1o). Bombesin receptors could be activated by bombesin peptides with C-termini in both amidated and nonamidated forms with different potencies. As described above, the C-terminal amide of the amidated NMB30 forms an additional hydrogen bond, which partially explains the phenomenon that the amidated bombesin peptides presented ~100-fold higher potencies than the nonamidated forms for their corresponding receptors (Supplementary information, Fig. S7k, l and Table S3). Superimposing the NMBR and GRPR complexes reveals that the binding modes between the two bombesin peptides are very similar, suggesting that they may activate bombesin receptors through a common mechanism (Supplementary information, Fig. S9a), although NMB30 and GRP(14–27) present different binding selectivity. The side chains of M10^N^ and M10^G^ insert into a conserved hydrophobic crevice at the bottom of the peptide-binding pocket and trigger the downwards rotameric movement of the toggle switch residue W^6.48^ in the conserved CWXP motif, facilitating the swing of F^6.44^ (Supplementary information, Fig. S9b). Following the repacking of an intrahelical contact between residues W^6.48^ and F^6.44^, the switching contacts of residues V^3.40^ towards W^6.48^ and L^5.51^ towards F^6.44^ contract the TM3–TM5–TM6 interface (Supplementary information, Fig. S9c), initiating the rotation of the cytoplasmic end of TM6. Overall, our study reveals the activation pathways of bombesin receptors and enriches the unique receptor-, ligand- and effector-specific activation pathways in the GPCR kingdom.

In summary, we present two cryo-EM structures of active G_q_-coupled NMBR and GRPR bound to NMB30 and GRP(14–27), respectively. Our structures depict the detailed interactions between the endogenous ligands and bombesin receptors. With the in-depth knowledge of ligand binding and selectivity of bombesin receptors, new opportunities may arise to design potent and efficacious modulators of bombesin receptors for the treatment of itch and other diseases.

## Supplementary information

Supplementary information

## Data Availability

Density maps and structure coordinates have been deposited in the Electron Microscopy Data Bank (EMDB) and the Protein Data Bank (PDB) with accession codes EMD-34413 and 8H0P for NMB30–NMBR–G_q_ complex; and EMD-34414 and 8H0Q for GRP(14–27)–GRPR–G_q_ complex.
