# Supplementary material for: Molecular recognition of itch-associated neuropeptides by bombesin receptors
Source: Cell Res. 2022 Nov 3;33(2):184–7. doi: 10.1038/s41422-022-00743-6 (PMC9892485; doi:10.1038/s41422-022-00743-6)
Supplement: Supplementary file 1 — Supplementary information [file 41422_2022_743_MOESM1_ESM.pdf]

Supplementary information

Materials and Methods

Constructs of NMBR, GRPR, and G proteins

Human NMBR (residues 1-361) and GRPR (residues 1-359) were cloned into pFastBac with an N-terminal haemagglutinin(HA) signal peptide followed by thermostabilized apocytochrome b_{562}RIL (BRIL)[1] as well as LgBiT at the C-terminal using homologous recombination (CloneExpress One Step Cloning Kit, Vazyme). After LgBiT, a TEV protease cleavage site and a double-MBP tag were added to both NMBR and GRPR constructs used for better expression and purification, and the point mutation L134$^{3.43}$Q of NMBR and L131$^{3.43}$Q of GRPR were generated[2]. The engineered Gα_q construct was generated based on mini-Gs/q71[3], which carries two dominant-negative mutations (corresponding to G203A and A326S)[4] to decrease the affinity of nucleotide-binding and facilitate the stability of Gαβγ complex. The N terminal 1–18 amino acids and the α-helical domain of the mini-$G_{s/q}$71 were substituted by the corresponding sequences of the human Gαi1 contributing to binding to the antibody fragments scFv16[5, 6]. Gβ1 was connected with a C-terminal SmBiT by a 15 residues linker. The engineered Gα_q, Gβ1-SmBiT and Gγ2 were cloned into pFastBac vectors independently (Invitrogen).

Expression and purification of NMBR/GRPR–G_q complex.

High Five cells (Expression systems) were cultured in ESF921 serum-free medium (Expression Systems) and infected with viruses of the receptor (NMBR or GRPR), Gα_q, Gβ1, Gγ2, and scFv16[7] in the ratio of 1:1:1:1:1 for 48 h at 27 ˚C using Bac-to-Bac baculovirus system. The cell pellets were lysed by dounce homogenization in a buffer containing 20 mM HEPES pH 7.4, 100 mM NaCl, 10 mM $MgCl_2$, 5 mM $CaCl_2$, 0.1 mM TCEP (Sigma-Aldrich), 10% glycerol, and EDTA-free protease inhibitor cocktail (TargetMol). The supernatant was isolated by centrifugation at 65,000 × g for 40 min to collect the membranes. The washed membranes were re-suspended in 20

mM HEPES pH 7.4, 100 mM NaCl, 10 mM $MgCl_2$, 5 mM $CaCl_2$, 10% glycerol, 10 μM peptide (NMB30 for NMBR and GRP (14-27) for GRPR, respectively, Synpeptide), 25 mU/mL apyrase (Sigma-Aldrich), 0.1 mM TCEP and EDTA-free protease inhibitor cocktail, and incubated at room temperature for 1.5 h. After incubation, 0.5% (w/v) n-dodecyl-β-d-maltopyranoside (DDM, Anatrace) and 0.1% (w/v) cholesteryl hemisuccinate TRIS salt (CHS, Anatrace) was used for solubilization at 4˚C for 2.5 h. The supernatant was collected by centrifugation at 65,000 × g for 40 min and then incubated with dextrin resin (Dextrin Beads 6FF, Smart Life Sciences) at 4˚C for 4 h. The resin was collected by centrifugation at 500 × g for 5 min, loaded onto a gravity flow column and washed with 10 column volumes of buffer containing 20 mM HEPES pH 7.4, 100 mM NaCl, 10 mM $MgCl_2$, 5 mM $CaCl_2$, 10% glycerol, 0.1 mM TCEP, 5 μM peptide, 0.05% (w/v) DDM and 0.01% (w/v) CHS. The detergent of washing buffer was then displaced by 0.1% (w/v) lauryl maltose neopentylglycol (LMNG, Anatrace) and 0.02% (w/v) CHS for 10 column volumes washing, followed by 0.03% (w/v) LMNG, 0.01% (w/v) glyco-diosgenin (GDN, Anatrace) and 0.008% (w/v) CHS for 20 column volumes washing. The protein was then treated with His-tagged TEV protease on column and further incubated at 4 ˚C for 8 h. The elution was concentrated with an Amicon Ultra Centrifugal Filter (MWCO 100 kDa) and injected onto a Superdex 200 increase 10/300 GL column (GE Healthcare) with running buffer 20 mM HEPES pH 7.4, 100 mM NaCl, 2 mM $MgCl_2$, 0.1 mM TCEP, 5 μM peptide, 0.00075%(w/v) LMNG, 0.00025% (w/v) GDN and 0.0002% (w/v) CHS. The fractions of monomeric protein complex for NMBR and GRPR were collected and evaluated by SDS-PAGE (Supplementary information, Fig. S2), and then concentrated by 30–50-fold for cryo-electron microscopy experiments.

Cryo-EM data collection

Cryo-EM grids were prepared with the Vitrobot Mark IV plunger (FEI) set to 4 ˚C and 100% humidity. Three-microliter of the NMB30-NMBR-G_q complex was applied to the glow discharged Au R1.2/1.3 holey carbon grids. The sample was incubated for 5 s

on the grids before blotting for 3 s (double-sided, blot force 2) and flash-frozen in liquid ethane immediately. The same condition was used for the sample GRP (14-27)-GRPR-G_q complex.

For NMB30-NMBR-G_q complex dataset, 4,858 movies were collected on a Titan Krios equipped with a Gatan K3 direct electron detection device at 300 kV with a magnification of 81,000, corresponding to a pixel size 1.04 Å. Image acquisition was performed with EPU Software (FEI Eindhoven, Netherlands). We collected a total of 36 frames accumulating to a total dose of 50 e^{-} Å^{-2} over 2.5 s exposure.

For GRP (14-27)-GRPR-G_q complex dataset, 9,002 movies were collected on a Titan Krios equipped with a Falcon4 direct electron detection device at 300 kV with a magnification of 96,000, corresponding to a pixel size 0.8 Å. Image acquisition was performed with EPU Software (FEI Eindhoven, Netherlands). We collected a total dose of 50 e^{-} Å^{-2} over 2.5 s exposure on each EER format movie[8]. Each movie was divided into 36 frames during motion correction.

Cryo-EM image processing

MotionCor2 was used to perform the frame-based motion-correction algorithm to generate drift-corrected micrograph for further processing and CTFFIND4 provided the estimation of the contrast transfer function (CTF) parameters[9, 10].

For NMB30-NMBR-G_q complex dataset, 480 aligned micrographs were deleted because of contaminations or bad ice quality. After selection, approximately 800 particles were manually picked and two-dimensional classes were calculated and used as references for automatic picking. All subsequent steps of particle picking, extraction, classification and post processing of refined models were performed with Relion3.0[11]. A total of 3,034,736 particles were extracted from the cryo-EM micrographs and followed by reference-free 2D classification, yielding 619,210

particles after clearance. Mask three-dimensional (3D) classification on the receptor part was used to separate out 355,509 particles that resulted to a clearer density of NMBR. We refined this portion of particles, which led to a structure at 3.52 Å global resolution. After CTF refinement, Bayesian polishing, and postprocessing with DeepEMhancer[12], then the particles were reconstituted to a 3.15 Å structure (Supplementary information, Fig. S3).

For GRP (14-27)-GRPR-G_q complex dataset, 762 aligned micrographs were deleted because of contaminations or bad ice quality. After selection, NMBR was used as 3D reference for automatic picking. All subsequent steps of particle picking, extraction, classification and post processing of refined models were performed with Relion3.0[11]. A total of 3,365,839 particles were extracted from the cryo-EM micrographs and followed by reference-free 2D classification, yielding 577,108 particles after clearance. Mask 3D classification on the receptor part was used to separate out 301,192 particles that resulted to a clearer density of GRPR. The second round of 3D classification was performed without mask and separated out 55,286 particles. We refined the remained particles, which led to a structure at 3.72 Å global resolution. After the postprocessing with DeepEMhancer[12], then the particles were reconstituted to a 3.3 Å structure (Supplementary information, Fig. S4).

Model building

NMBR and GRPR structures predicted from Alphafold2 were used as the starting reference models for receptors building[13]. Structures of $G\alpha_q$, Gβ, Gγ and the scFv16 were derived from PDB entry 7WKD (unpublished) were rigid body fit into the density. All models were fitted into the EM density map using UCSF Chimera[14] followed by iterative rounds of manual adjustment and automated rebuilding in COOT[15] and PHENIX[16], respectively. The model was finalized by rebuilding in ISOLDE[17] followed by refinement in PHENIX with torsion-angle restraints to the input model. The final

model statistics were validated using Comprehensive validation (cryo-EM) in PHENIX[16] and provided in the supplementary information (Supplementary information, Table S1). All structural figures were prepared using Chimera[14], Chimera X[18], and PyMOL (Schrödinger, LLC.).

Function essay

AD293 cells (Agilent) were cultured in DMEM/high Glucose medium (GE healthcare) supplemented with 10% (v/v) fetal bovine serum (FBS, Gemini) and 1% penicillin/streptomycin and maintained at 37°C in 5% CO_2 incubator. Inositol phosphate 1 (IP1) production was measured using the IP-One HTRF kit (Cisbio, 621PAPEJ)[19]. Briefly, cells were seeded onto 12-well cell culture plates for 16 h before transfection. The cells were then transiently with different NMBR or GRPR constructs using FuGENE HD transfection reagent. After 24 h, cells were harvested and resuspended in IP1 stimulation buffer at a density of 7×10^5 cells/mL. Cells were then plated onto 384-well assay plates at 4900 cells/7 μL/well. Another 7 μL IP1 Stimulation Buffer 2 containing ligand was added to the cells, and the incubation lasted for 1 h at 37 °C. Intracellular IP1 measurement was carried with the IP-One HTRF kit and EnVision multiplate reader according to the manufacturer's instructions. The HTRF ratio was converted to a response (%) using the following formula: response (%) = ratio of sample/WT×100. Data presented are mean±S.E.M. of at least three biologically independent experiments.

Cell-surface expression assay

Cell-surface expression for each NMBR and GRPR mutant was monitored by a fluorescence-activated cell sorting (FACS) assay. The mutants were cloned into pcDNA6.0 vector (Invitrogen) with a N-terminal FLAG tag. The cell seeding and transfection follow the same method as function assay. After 24h of transfection, cells were washed once with PBS and digested with 0.2% (w/v) EDTA in PBS. Thereafter, the expressed cells were incubated with Monoclonal anti-FLAG M2-FITC (Sigma-

Aldrich) at a dilution of 1:100 for 15 min at 4 °C, and then a 9-fold excess of PBS was added to cells. After cells were resuspended, fluorescence intensity was quantified in a BD Accuri C6 flow cytometer system (BD Biosciences) at excitation 488 nm and emission 519 nm. The FACS data were analyzed by BD Accuri C6 software 1.0.264.21 and data were normalized to WT.

Data Resources

Materials are available from the corresponding authors upon reasonable request. Density maps and structure coordinates have been deposited in the Electron Microscopy Data Bank (EMDB) and the Protein Data Bank (PDB) with accession codes EMD-34413 and 8H0P for NMB30-NMBR-Gq complex; and EMD-34414 and 8H0Q for GRP (14-27)-GRPR-Gq complex. Source data are provided with this paper.

References

1 Chun E, Thompson AA, Liu W *et al.* Fusion Partner Toolchest for the Stabilization and Crystallization of G Protein-Coupled Receptors. *Structure* 2012; **20**:967-976.

2 Ceraudo E, Horioka M, Mattheisen JM *et al.* Direct evidence that the GPCR CysLTR2 mutant causative of uveal melanoma is constitutively active with highly biased signaling. *J Biol Chem* 2021; **296**:100163.

3 Nehme R, Carpenter B, Singhal A *et al.* Mini-G proteins: Novel tools for studying GPCRs in their active conformation. *PLoS One* 2017; **12**:e0175642.

4 Liu P, Jia MZ, Zhou XE *et al.* The structural basis of the dominant negative phenotype of the Galphai1beta1gamma2 G203A/A326S heterotrimer. *Acta Pharmacol Sin* 2016; **37**:1259-1272.

5 Maeda S, Qu Q, Robertson MJ, Skiniotis G, Kobilka BK. Structures of the M1 and M2 muscarinic acetylcholine receptor/G-protein complexes. *Science* 2019; **364**:552-557.

6 Kang Y, Kuybeda O, de Waal PW *et al.* Cryo-EM structure of human rhodopsin bound to an inhibitory G protein. *Nature* 2018; **558**:553-558.

7 Maeda S, Koehl A, Matile H *et al.* Development of an antibody fragment that stabilizes GPCR/G-protein complexes. *Nat Commun* 2018; **9**:3712.

8 Guo H, Franken E, Deng Y *et al.* Electron-event representation data enable efficient cryoEM file storage with full preservation of spatial and temporal resolution. *IUCrJ* 2020; **7**:860-869.

9 Zheng SQ, Palovcak E, Armache JP, Verba KA, Cheng Y, Agard DA. MotionCor2: anisotropic correction of beam-induced motion for improved cryo-electron microscopy. *Nat Methods* 2017; **14**:331-332.

10 Rohou A, Grigorieff N. CTFFIND4: Fast and accurate defocus estimation from electron micrographs. *J Struct Biol* 2015; **192**:216-221.

11 Zivanov J, Nakane T, Forsberg BO *et al.* New tools for automated high-resolution cryo-EM structure determination in RELION-3. *Elife* 2018; **7**.

12 Sanchez-Garcia R, Gomez-Blanco J, Cuervo A, Carazo JM, Sorzano COS, Vargas J. DeepEMhancer: a deep learning solution for cryo-EM volume post-processing. *Commun Biol* 2021; **4**:874.

13 Tunyasuvunakool K, Adler J, Wu Z *et al.* Highly accurate protein structure prediction for the human proteome. *Nature* 2021; **596**:590-596.

14 Pettersen EF, Goddard TD, Huang CC *et al.* UCSF Chimera--a visualization system for exploratory research and analysis. *J Comput Chem* 2004; **25**:1605-1612.

15 Emsley P, Cowtan K. Coot: model-building tools for molecular graphics. *Acta Crystallogr D Biol Crystallogr* 2004; **60**:2126-2132.

16 Adams PD, Gopal K, Grosse-Kunstleve RW *et al.* Recent developments in the PHENIX software for automated crystallographic structure determination. *J Synchrotron Radiat* 2004; **11**:53-55.

17 Croll TI. ISOLDE: a physically realistic environment for model building into low-resolution electron-density maps. *Acta Crystallogr D Struct Biol* 2018; **74**:519-530.

18 Pettersen EF, Goddard TD, Huang CC *et al.* UCSF ChimeraX: Structure visualization for researchers, educators, and developers. *Protein Sci* 2021; **30**:70-82.

19 Leyris JP, Roux T, Trinquet E *et al.* Homogeneous time-resolved fluorescence-based assay to screen for ligands targeting the growth hormone secretagogue receptor type 1a. *Anal Biochem* 2011; **408**:253-262.

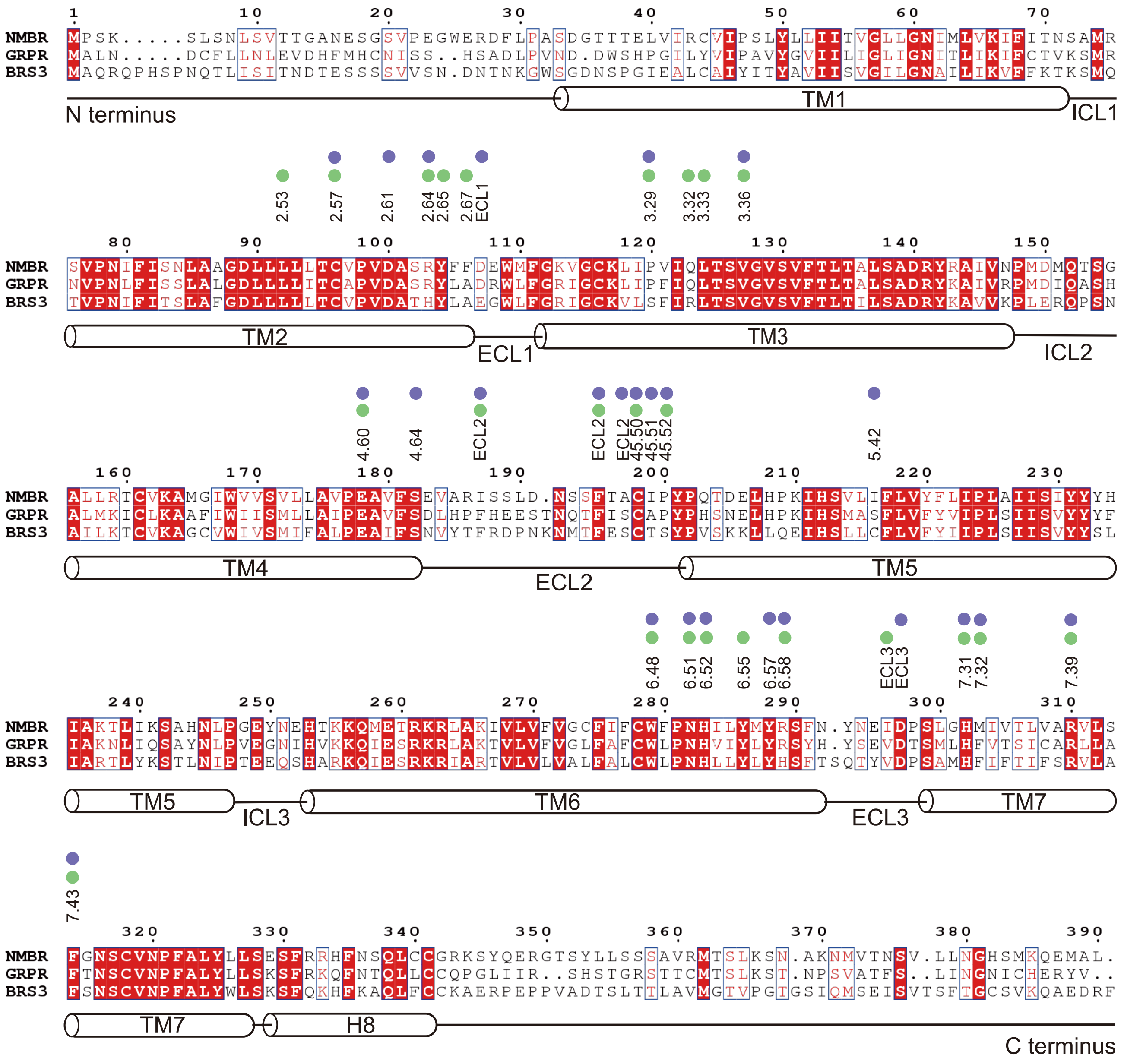

NMBR
GRPR
BRS3
N terminus
TM1
ICL1
TM2
ECL1
TM3
ICL2
TM4
ECL2
TM5
ICL3
TM6
ECL3
TM7
H8
C terminus

Supplementary information, Fig. S1 Sequence alignment of NMBR, GRPR and BRS3. Secondary structure elements are annotated underneath the sequences based on the structure of the NMB30-NMBR-G_q complex. The purple and green solid circle represent the ligand binding sites of NMBR and GRPR, respectively.

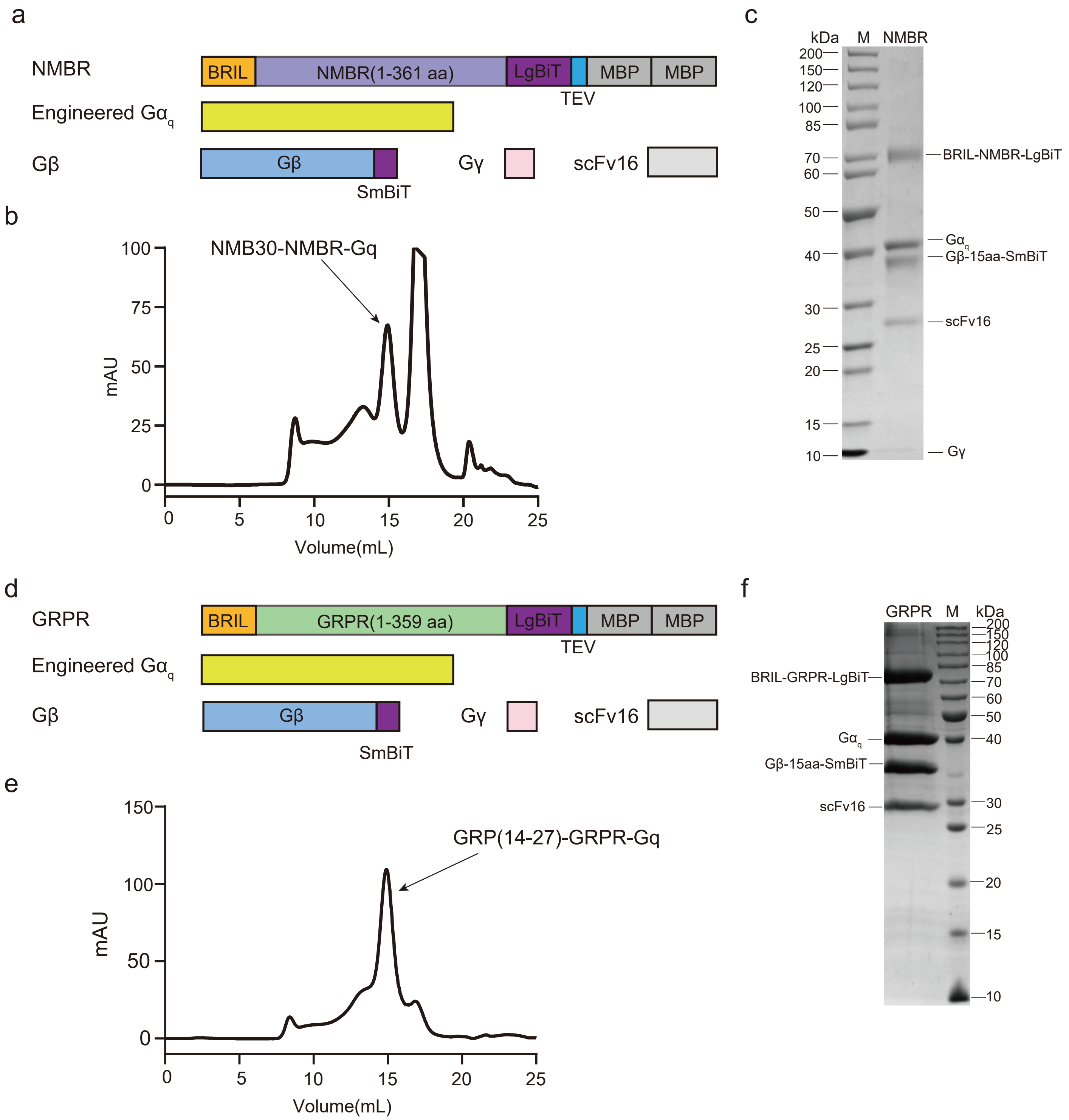
a
NMBR
BRIL
NMBR(1-361 aa)
LgBiT
MBP
MBP
TEV
Engineered Gαq
Gβ
Gβ
SmBiT
Gγ
scFv16
b
NMB30-NMBR-Gq
mAU
Volume(mL)
c
kDa
M
NMBR
BRIL-NMBR-LgBiT
Gαq
Gβ-15aa-SmBiT
scFv16
Gγ
d
GRPR
BRIL
GRPR(1-359 aa)
LgBiT
MBP
MBP
TEV
Engineered Gαq
Gβ
Gβ
SmBiT
Gγ
scFv16
e
GRP(14-27)-GRPR-Gq
mAU
Volume(mL)
f
GRPR
M
kDa
BRIL-GRPR-LgBiT
Gαq
Gβ-15aa-SmBiT
scFv16

Supplementary information, Fig. S2 Purification and characterization of NMB30-NMBR–G_q complex and GRP (14-27)-GRPR-G_q complex.

a Schematic diagram of the protein engineering of NMBR, engineered $G\alpha_q$, Gβ, Gγ and scFv16 used in this study.

b Gel filtration (Superdex 200 Increase 10/300 column, GE Healthcare) profile of the NMB30-NMBR–G_q complex. The black arrow indicates the sharp peak for NMB30-NMBR–G_q complex.

c Coomassie-stained SDS-PAGE analysis of the purified NMB30-NMBR–G_q complex, showing balanced ratios for each subunit.

d Schematic diagram of the protein engineering of NMBR, engineered $G\alpha_q$, Gβ, Gγ and scFv16 used in this study.

e Gel filtration (Superdex 200 Increase 10/300 column, GE Healthcare) profile of the GRP (14-27)-GRPR-G_q complex. The black arrow indicates the sharp peak for GRP (14-27)-GRPR-G_q complex.

f Coomassie-stained SDS-PAGE analysis of the purified GRP (14-27)-GRPR-G_q complex, showing balanced ratios for certain subunits, except for the Gγ subunit.

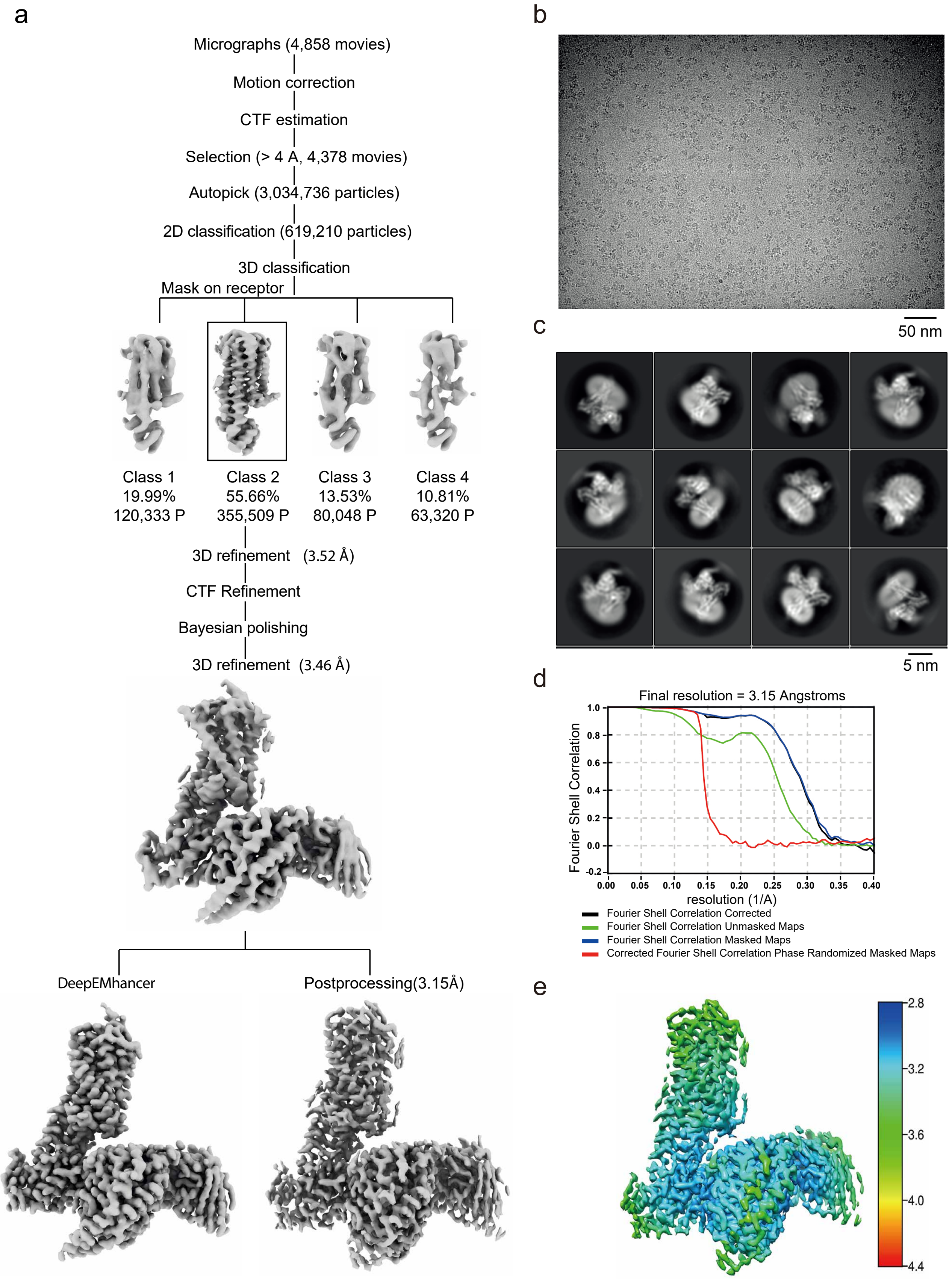
a
Micrographs (4,858 movies)
Motion correction
CTF estimation
Selection (> 4 A, 4,378 movies)
Autopick (3,034,736 particles)
2D classification (619,210 particles)
3D classification
Mask on receptor
Class 1
19.99%
120,333 P
Class 2
55.66%
355,509 P
Class 3
13.53%
80,048 P
Class 4
10.81%
63,320 P
3D refinement (3.52 Å)
CTF Refinement
Bayesian polishing
3D refinement (3.46 Å)
DeepEMhancer
Postprocessing(3.15Å)
b
50 nm
c
5 nm
d
Final resolution = 3.15 Angstroms
Fourier Shell Correlation
resolution (1/A)
Fourier Shell Correlation Corrected
Fourier Shell Correlation Unmasked Maps
Fourier Shell Correlation Masked Maps
Corrected Fourier Shell Correlation Phase Randomized Masked Maps
e
2.8
3.2
3.6
4.0
4.4

Supplementary information, Fig. S3 Cryo-EM data processing of NMB30-NMBR-G_q complex.

a Flowchart of computational sorting of cryo-EM data.

b A representative cryo-EM micrograph of NMB30-NMBR–Gq complex with 50 nm scale bar included as a size reference.

c Twelve representative reference-free 2D cryo-EM class averages. Scale bar, 5 nm.

d ‘Gold-standard’ Fourier shell correlation curve of the reconstruction. The resolution was reported at 3.15 Å using the Fourier shell cutoff at 0.143.

e Local resolution map of NMB30-NMBR–G_q complex.

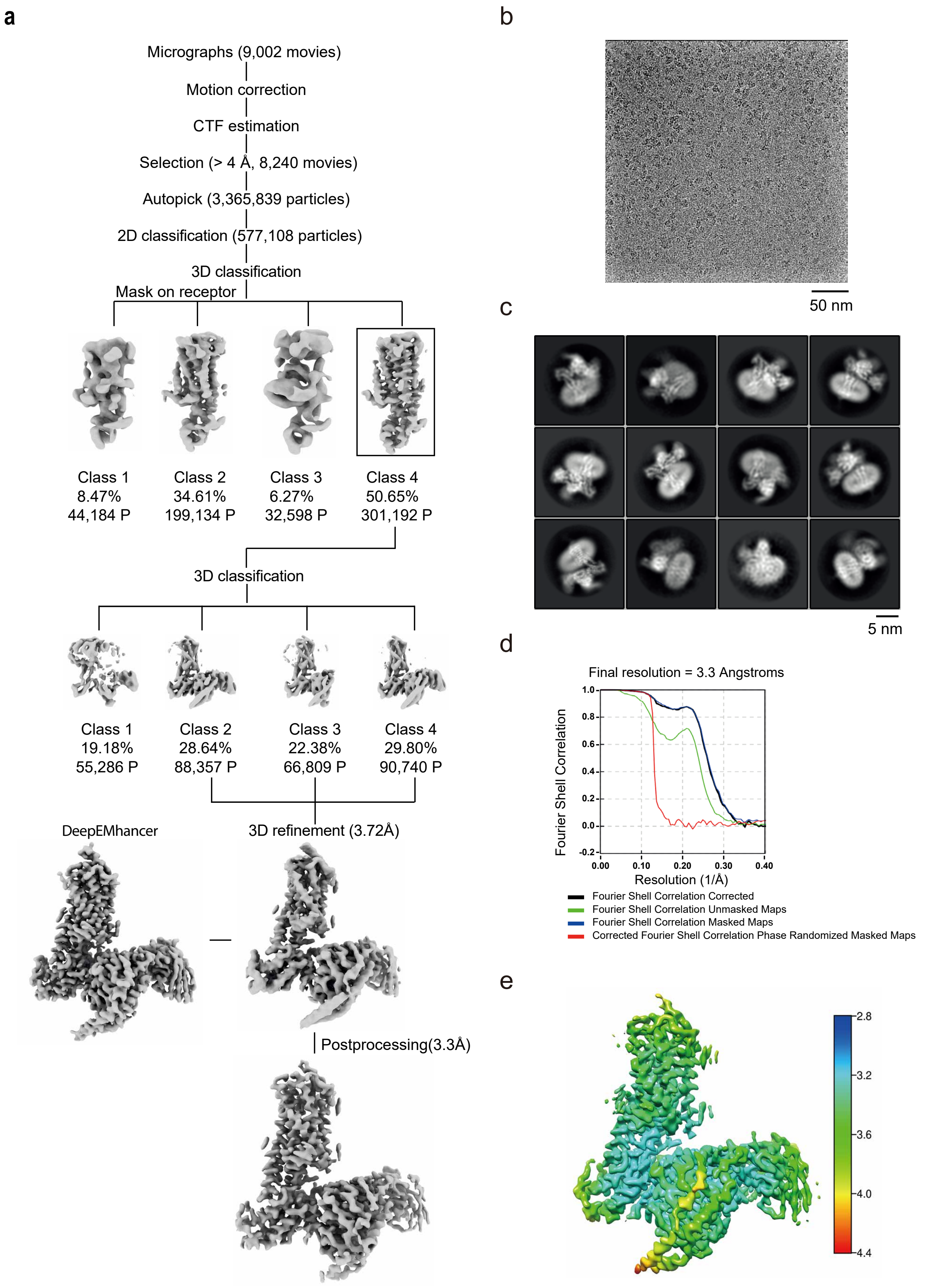
a
Micrographs (9,002 movies)
Motion correction
CTF estimation
Selection (> 4 Å, 8,240 movies)
Autopick (3,365,839 particles)
2D classification (577,108 particles)
3D classification
Mask on receptor
Class 1 8.47% 44,184 P
Class 2 34.61% 199,134 P
Class 3 6.27% 32,598 P
Class 4 50.65% 301,192 P
3D classification
Class 1 19.18% 55,286 P
Class 2 28.64% 88,357 P
Class 3 22.38% 66,809 P
Class 4 29.80% 90,740 P
DeepEMhancer
3D refinement (3.72Å)
Postprocessing(3.3Å)
b
50 nm
c
5 nm
d
Final resolution = 3.3 Angstroms
Fourier Shell Correlation
Resolution (1/Å)
Fourier Shell Correlation Corrected
Fourier Shell Correlation Unmasked Maps
Fourier Shell Correlation Masked Maps
Corrected Fourier Shell Correlation Phase Randomized Masked Maps
e
2.8
3.2
3.6
4.0
4.4

Supplementary information, Fig. S4 Cryo-EM data processing of GRP (14-27)-GRPR-G_q complex.

a Flowchart of computational sorting of cryo-EM data.

b A representative cryo-EM micrograph of GRP (14-27)-GRPR-G_q complex with 50 nm scale bar included as a size reference.

c Twelve representative reference-free 2D cryo-EM class averages. Scale bar, 5 nm.

d Gold-standard' Fourier shell correlation curve of the reconstruction. The resolution was reported at 3.3 Å using the Fourier shell cutoff at 0.143.

e Local resolution map of GRP (14-27)-GRPR-G_q complex.

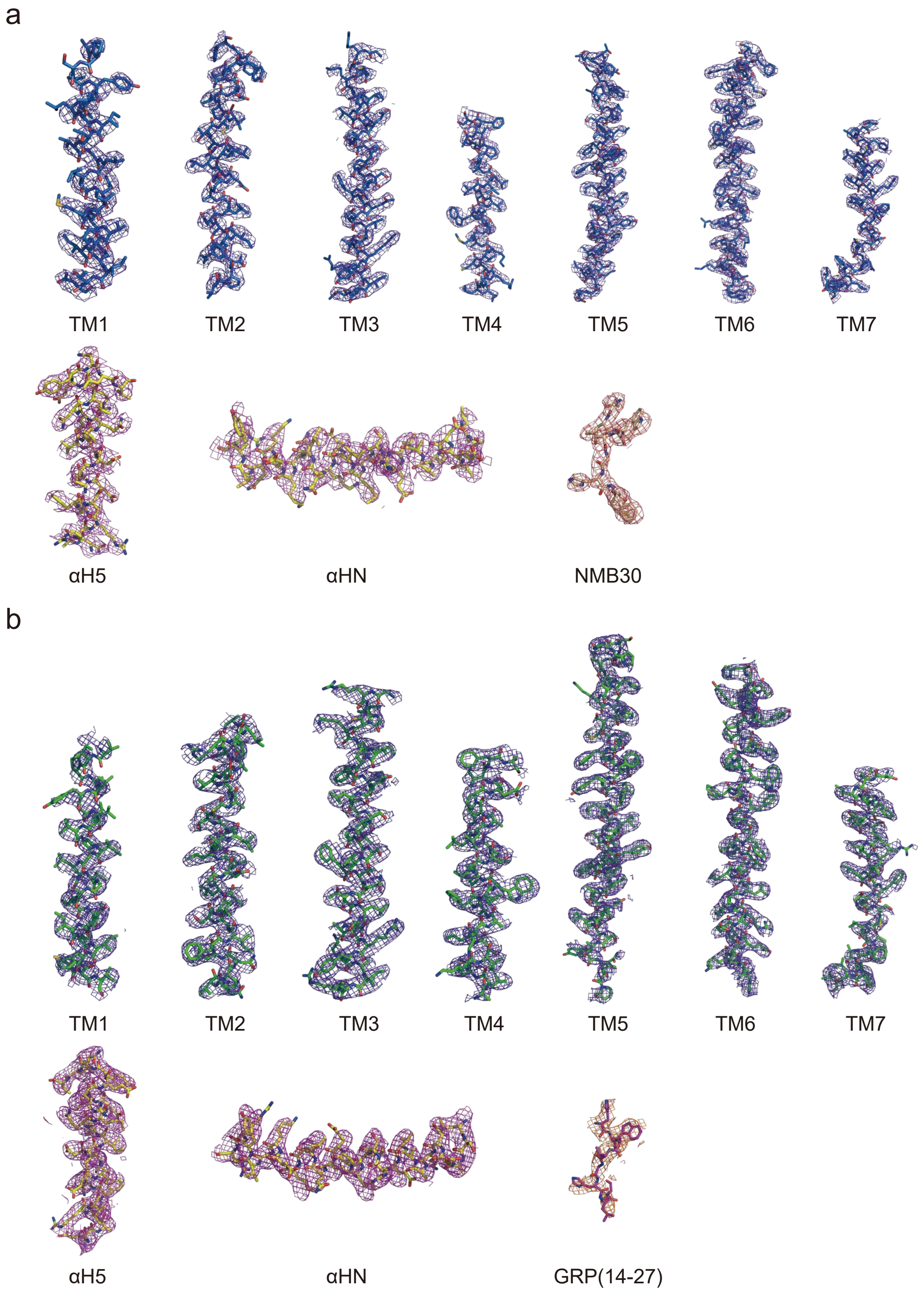
a
TM1
TM2
TM3
TM4
TM5
TM6
TM7
αH5
αHN
NMB30
b
TM1
TM2
TM3
TM4
TM5
TM6
TM7
αH5
αHN
GRP(14-27)

Supplementary information, Fig. S5 Cryo-EM density maps of TM1-7 in receptors, αH5 and αHN in G_q proteins, and ligands in NMBR structure (a) and GRPR structure (b).

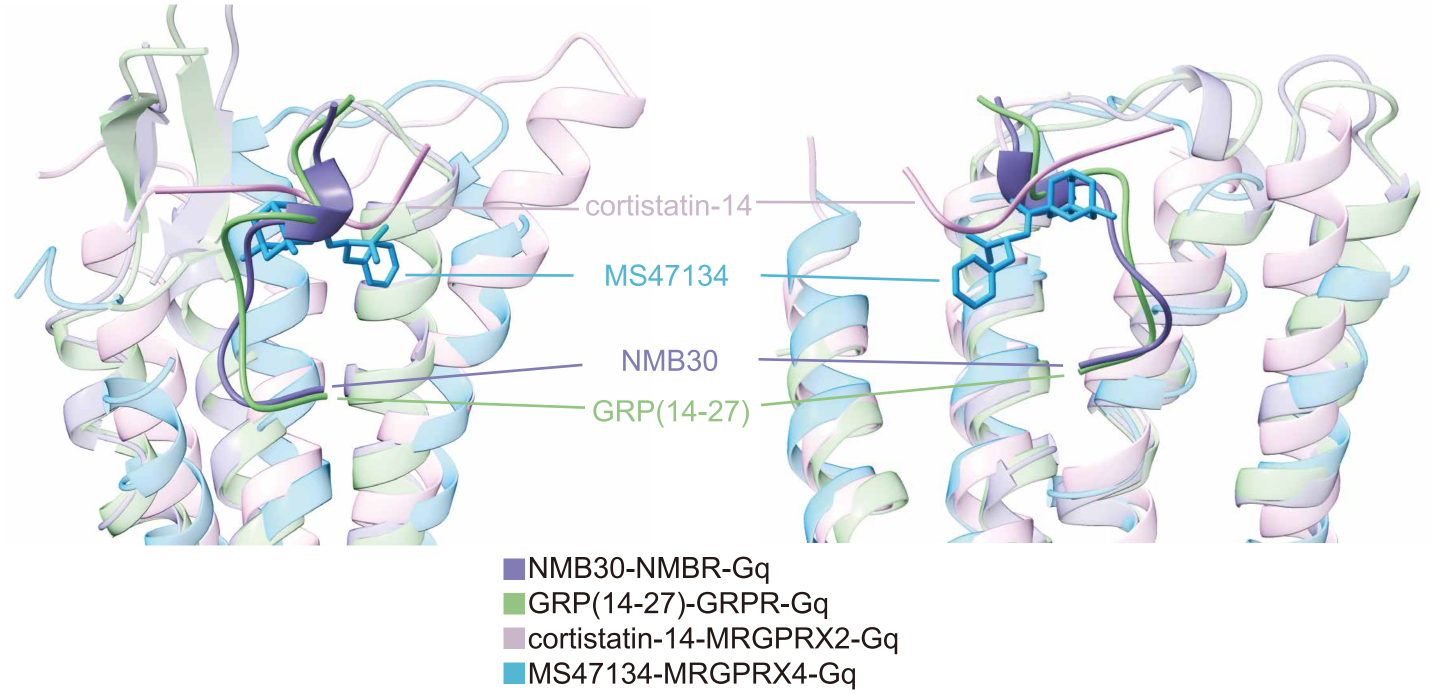
cortistatin-14
MS47134
NMB30
GRP(14-27)
NMB30-NMBR-Gq
GRP(14-27)-GRPR-Gq
cortistatin-14-MRGPRX2-Gq
MS47134-MRGPRX4-Gq

Supplementary information, Fig. S6 Structural comparisons of our bombesin receptors with the peptide bound MRGPRX2/4, showing the differences in the ligand-binding pockets.

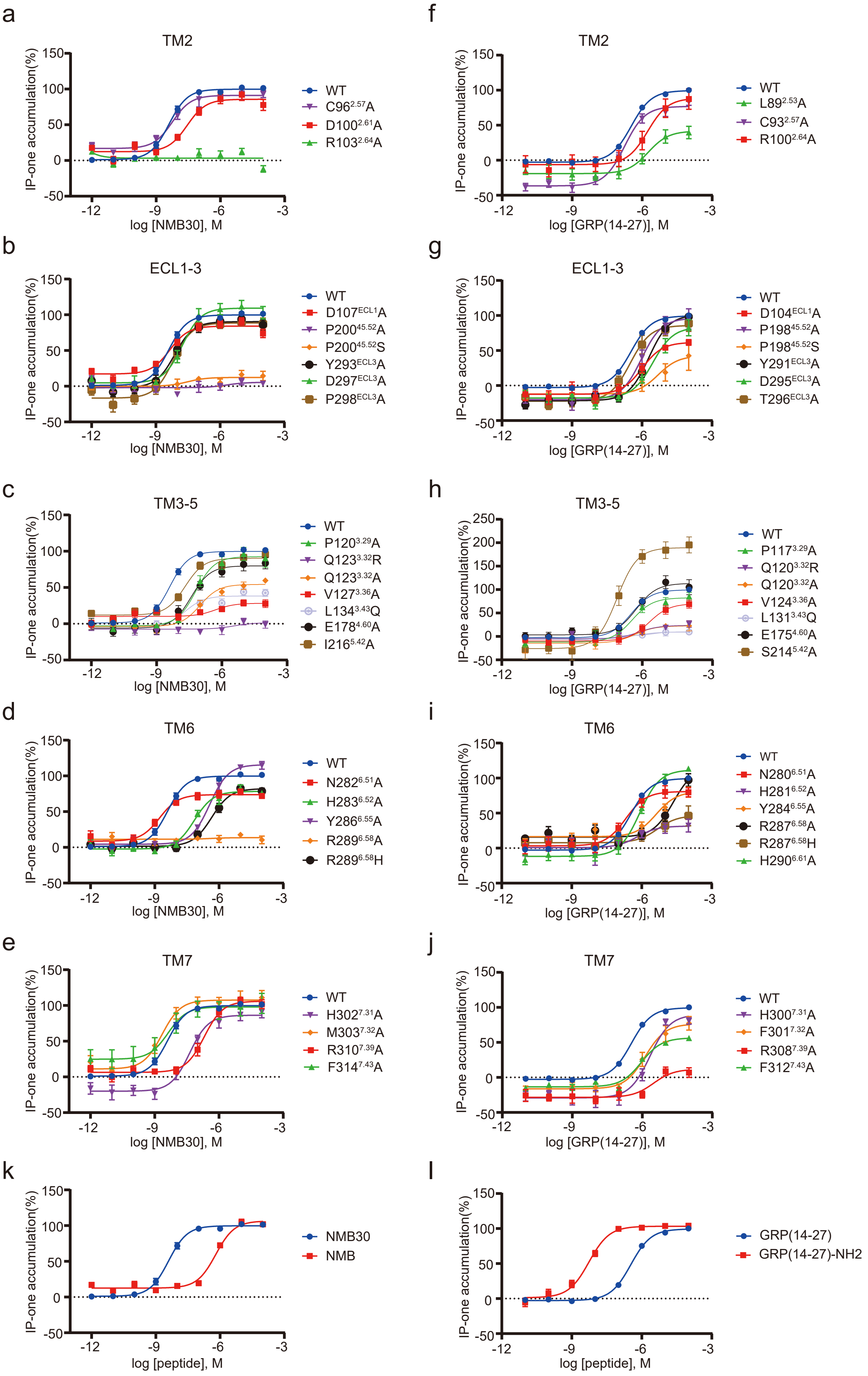
a
TM2
IP-one accumulation(%)
log [NMB30], M
WT
C96$^{2.57}$A
D100$^{2.61}$A
R103$^{2.64}$A
b
ECL1-3
IP-one accumulation(%)
log [NMB30], M
WT
D107^{ECL1}A
P200$^{45.52}$A
P200$^{45.52}$S
Y293^{ECL3}A
D297^{ECL3}A
P298^{ECL3}A
c
TM3-5
IP-one accumulation(%)
log [NMB30], M
WT
P120$^{3.29}$A
Q123$^{3.32}$R
Q123$^{3.32}$A
V127$^{3.36}$A
L134$^{3.43}$Q
E178$^{4.60}$A
I216$^{5.42}$A
d
TM6
IP-one accumulation(%)
log [NMB30], M
WT
N282$^{6.51}$A
H283$^{6.52}$A
Y286$^{6.55}$A
R289$^{6.58}$A
R289$^{6.58}$H
e
TM7
IP-one accumulation(%)
log [NMB30], M
WT
H302$^{7.31}$A
M303$^{7.32}$A
R310$^{7.39}$A
F314$^{7.43}$A
f
TM2
IP-one accumulation(%)
log [GRP(14-27)], M
WT
L89$^{2.53}$A
C93$^{2.57}$A
R100$^{2.64}$A
g
ECL1-3
IP-one accumulation(%)
log [GRP(14-27)], M
WT
D104^{ECL1}A
P198$^{45.52}$A
P198$^{45.52}$S
Y291^{ECL3}A
D295^{ECL3}A
T296^{ECL3}A
h
TM3-5
IP-one accumulation(%)
log [GRP(14-27)], M
WT
P117$^{3.29}$A
Q120$^{3.32}$R
Q120$^{3.32}$A
V124$^{3.36}$A
L131$^{3.43}$Q
E175$^{4.60}$A
S214$^{5.42}$A
i
TM6
IP-one accumulation(%)
log [GRP(14-27)], M
WT
N280$^{6.51}$A
H281$^{6.52}$A
Y284$^{6.55}$A
R287$^{6.58}$A
R287$^{6.58}$H
H290$^{6.61}$A
j
TM7
IP-one accumulation(%)
log [GRP(14-27)], M
WT
H300$^{7.31}$A
F301$^{7.32}$A
R308$^{7.39}$A
F312$^{7.43}$A
k
IP-one accumulation(%)
log [peptide], M
NMB30
NMB
l
IP-one accumulation(%)
log [peptide], M
GRP(14-27)
GRP(14-27)-NH2

Supplementary information, Fig. S7 IP1 response curves of NMBR and GRPR. Effects of NMBR mutations (**a-e**) or GRPR mutations (**f-j**) on NMB30 or GRP (14-27)-induced IP1 production. Effects of amidated and non-amidated forms of NMB30 or GRP (14-27) on receptor activation (**k, l**). Data are presented as mean ± S.E.M. of at least three independent experiments. Source data are available online.

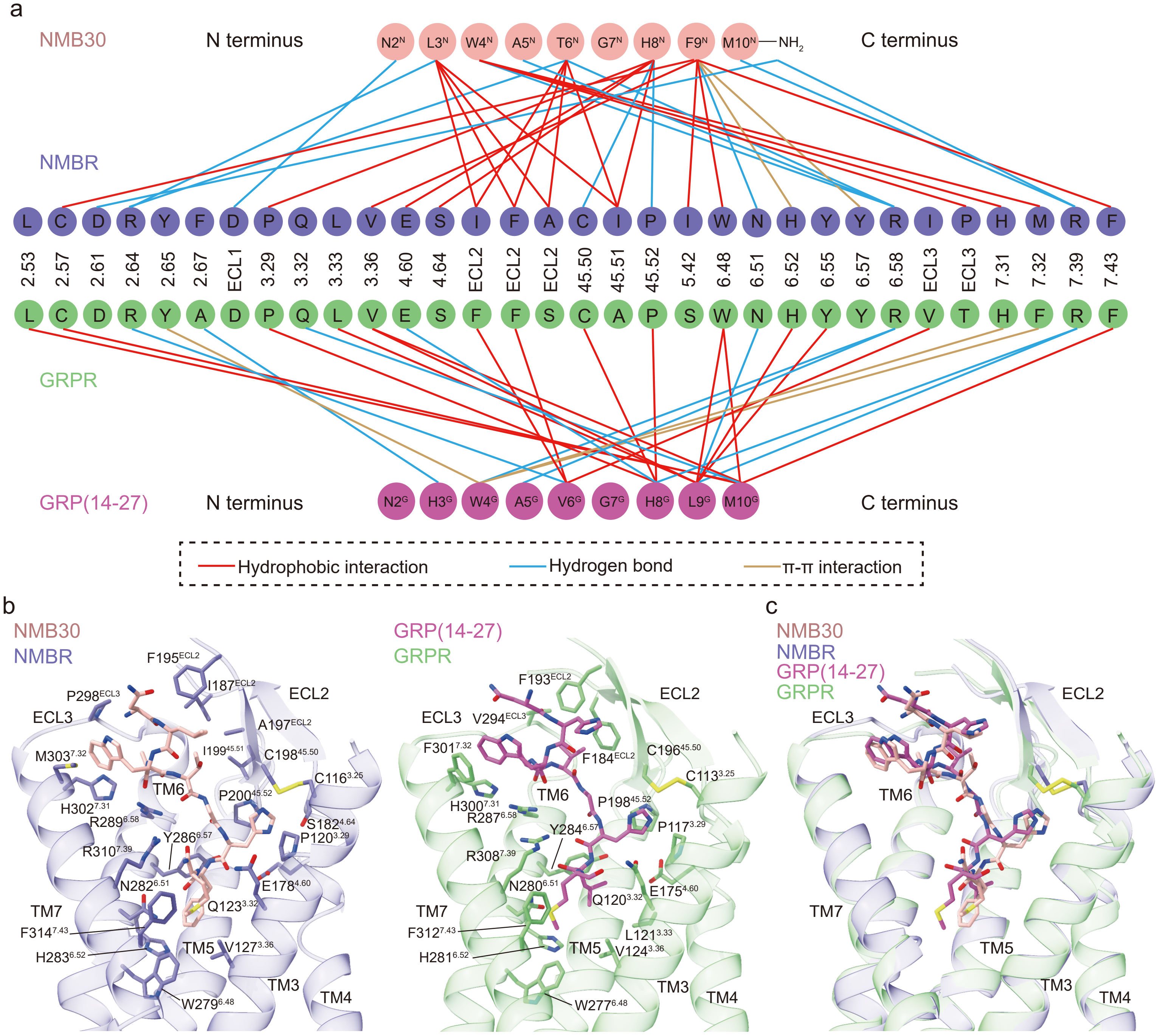

a
NMB30
N terminus
N2N L3N W4N A5N T6N G7N H8N F9N M10N NH2
C terminus
NMBR
GRPR
GRP(14-27)
N terminus
N2G H3G W4G A5G V6G G7G H8G L9G M10G
C terminus
Hydrophobic interaction
Hydrogen bond
π-π interaction
b
NMB30
NMBR
GRP(14-27)
GRPR
ECL2
ECL3
TM3
TM4
TM5
TM6
TM7
c
NMB30
NMBR
GRP(14-27)
GRPR

Supplementary information, Fig. S8 Comparison of interactions between NMB30 and GRP (14-27) with two bombesin receptors.

a Representative interaction network of NMB30 bound to NMBR and GRP (14-27) bound to GRPR. Amino acids in NMB30 and GRP (14-27), as well as residues in corresponding binding pockets of NMBR and GRPR, are displayed as circled one-letter codes. Lines show interactions between peptides and bombesin receptor subtypes.

b The binding pockets of NMB30 and GRP (14-27) in receptors NMBR and GRPR.

c Superposition of the binding pockets of NMB30 and GRP (14-27). For (**b**) and (**c**), the ligands and receptors are presented as sticks and cartoon, respectively.

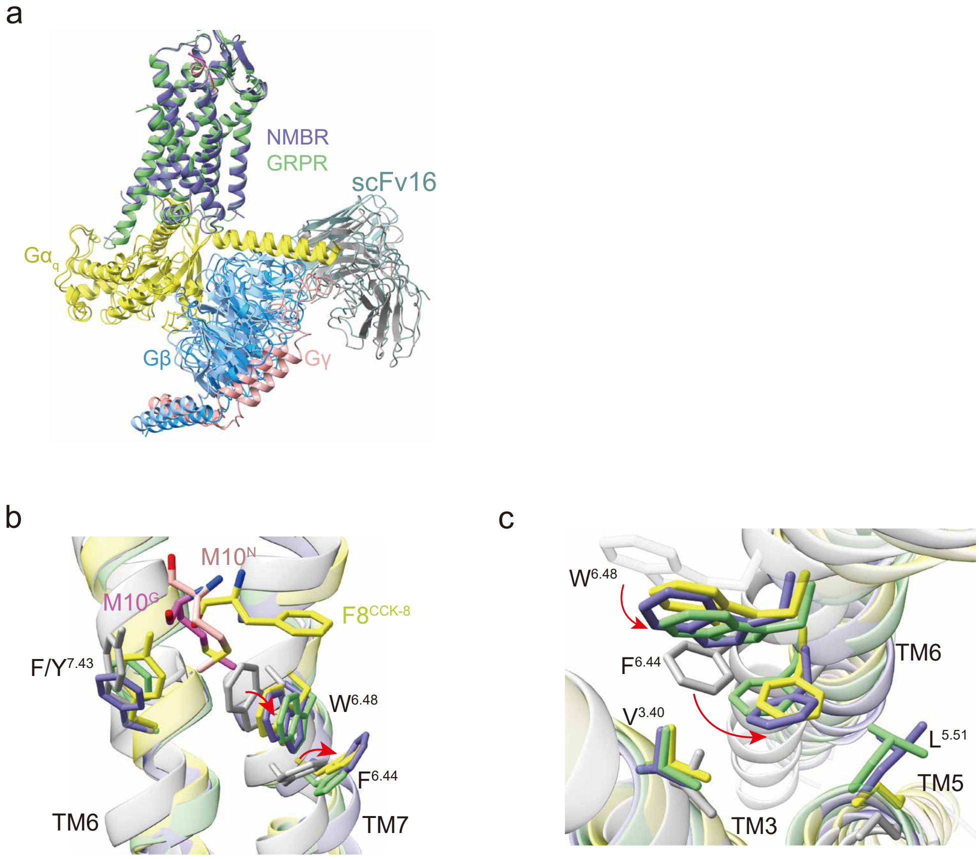
a
NMBR
GRPR
scFv16
Gαq
Gβ
Gγ
b
M10N
M10G
F8CCK-8
F/Y7.43
W6.48
F6.44
TM6
TM7
c
W6.48
F6.44
TM6
V3.40
L5.51
TM5
TM3

Supplementary information, Fig. S9 Structural alignment of the structures of the NMB30-NMBR-G_q complex and GRP (14-27)-GRPR-G_q complex(a) and molecular basis for activation of NMBR and GRPR (b and c). Conformational changes of the conserved 'micro-switches' upon receptor activation, including toggle switch (**b**), transmission switch consisting of $L^{5.51}$ -$F^{6.44}$ and $V^{3.40}$- $W^{6.48}$(**c**)

Supplementary information, Table S1 Cryo-EM data collection, model refinement and validation statistics.

	NMB30-NMBR-G_q complex	GRP(14-27)-GRPR-G_q complex
**Data collection and processing**		
Magnification	81,000	96,000
Voltage (kV)	300	300
Electron exposure ($e^–/Å^2$)	50	50
Defocus range (μm)	-1.0~-3.0	-1.0~-3.0
Pixel size (Å)	1.04	0.8
Symmetry imposed	C1	C1
Initial particle projections (no.)	3,034,736	3,365,839
Final particle projections (no.)	355,509	245,906
Map resolution (Å)	3.15	3.30
Map resolution range (Å)	3.0-4.5	3.0-4.5
FSC threshold	0.143	0.143
**Model Refinement**		
Refinement package	PHENIX-1.17.1-3660	PHENIX-1.17.1-3660
Real or reciprocal space	Real space	Real space
Model-Map CC (mask)	0.65	0.72
Model resolution (Å)	3.42	3.89
FSC threshold	0.5	0.5
B factors (Å2, mean value)		
Protein residues	29.72	104.30
Ligands	-	20.00
**Model composition**		
Non-hydrogen atoms	8,932	8,950
Protein residues	1,137	1,134
R.m.s. deviations		
Bond lengths (Å)	0.001	0.001
Bond angles (°)	0.385	0.470
**Validation**		
MolProbity score	1.31	1.43
Clashscore	5.68	7.81
Rotamer outliers (%)	0.10	0.21
Ramachandran plot		
Favored (%)	98.83	99.01
Allowed (%)	1.17	0.99
Disallowed (%)	0.00	0.00
**Data availability**		
EMDB entry	EMD-34413	EMD-34414
PDB entry	8H0P	8H0Q

Supplementary information, Table S2 Amino acid sequences of Bn-related peptides used or mentioned in this study.

Peptide name	Bombesin analogue	N-Terminus	position relative to Bn													
			1	2	3	4	5	6	7	8	9	10	11	12	13	14
	Bombesin-related															
Bn	Bombesin		pE	Q	R	L	G	N	Q	W	A	V	G	H	L	$M-NH_2$
GRP27	GRP (1–27)	VPLPAGGGTVLTK	M	Y	P	R	G	N	H	W	A	V	G	H	L	$M-NH_2$
GRP (14–27)	GRP (14–27)		M	Y	P	R	G	N	H	W	A	V	G	H	L	M
Aly	Alytesin			G	R	L	G	T	Q	W	A	V	G	H	L	$M-NH_2$
NMC	Neuromedin C						G	N	H	W	A	V	G	H	L	$M-NH_2$
	Neuromedin B-related															
NMB	Neuromedin B						G	N	L	W	A	T	G	H	F	$M-NH_2$
NMB30	Neuromedin B (1-30)	LSWDLPEPRSRASKIR	V	H	R	R	G	N	L	W	A	T	G	H	F	$M-NH_2$
Roh-Lit	Rohdei-litorin							pE	L	W	A	T	G	H	F	$M-NH_2$
Lit	Litorin							pE	Q	W	A	V	G	H	F	$M-NH_2$
Ran	Ranatensin					pE	V	P	Q	W	A	V	G	H	F	$M-NH_2$
	Phyllolitorin-related															
PLL	Phyllolitorin							pE	L	W	A	V	G	S	F	$M-NH_2$
LeuPLL	[Leu8]phyllolitorin							pE	L	W	A	V	G	S	L	$M-NH_2$
							1	2	3	4	5	6	7	8	9	10
							position relative to NMB or NMC									

Supplementary information, Table S3 Ligand binding affinities and expression levels of WT and mutated NMBR and GRPR. The wild type (WT) and mutants of NMBR and GRPR discussed in this manuscript were individually analyzed. The affinities are derived from at least 3 independent experiments using IP1 function assay. The expression level of mutant NMBR and GRPR were normalized to wild-type NMBR and GRPR as 100%, respectively. Each data point represents mean ± standard error of the mean (S.E.M.). All data were analyzed by two-sided Student's t test. *P<0.05, **P<0.01, ***P<0.001 vs. WT. Source data are available online. Definitions: NA – not applicable; NT, not tested.

Residue Number	NMBR mutant	pEC_{50}±S.E.M.	Emax± S.E.M.(%WT)	Expression (% of WT)	GRPR mutant	pEC_{50}±S.E.M.	Emax± S.E.M.(%WT)	Expression (% of WT)
-	WT	8.44±0.14	100	100±4.14	WT	6.45±0.07	100	100±29.335
2.53	L92A	NT	NT	NT	L89A	5.97±0.30	41.31±18.00	155.57±28.22
2.57	C96A	8.25±0.09	91.15±8.01	74.71±3.79	C93A	6.78±0.07*	135.42±62.17	262.60±20.67
2.61	D100A	7.58±0.13**	85.81±12.88	53.52±7.35	D97A	NT	NT	NT
2.64	R103A	NA	NA	90.69±3.02	R100A	5.72±0.11**	88.66±25.73	170.49±22.41
ECL1	D107A	8.44±0.16	84.17±10.41	68.84±3.05	D104A	5.87±0.26	63.70±1.46**	85.32±7.97
3.29	P120A	7.21±0.04***	92.66±20.11	120.21±7.57	P117A	6.37±0.21	128.81±59.52	346.31±20.86
3.32	Q123R	NA	NA	40.12±3.99	Q120R	NA	NA	NT
3.32	Q123A	7.05±0.14***	54.21±5.36*	7.15±0.24	Q120A	NA	NA	13.09±3.43
3.36	V127A	NA	NA	24.72±5.30	V124A	5.65±0.16*	94.37±34.90	184.24±25.85
3.43	L134Q	7.70±0.15*	38.33±9.00*	11.81±0.73	L131Q	NA	NA	128.07±58.55
4.60	E178A	7.43±0.18**	80.05±13.17	73.16±1.90	E175A	6.28±0.08	113.07±16.97	155.24±5.12
45.52	P200A	NA	NA	59.29±1.05	P198A	6.00±0.09*	96.52±26.70	156.74±16.41
45.52	P200S	NA	NA	43.96±7.55	P198S	5.31±0.07***	45.27±39.47	94.46±6.80
5.42	I216A	7.70±0.03**	90.37±6.84	14.71±1.51	S214A	7.08±0.09**	189.80±29.77	185.15±21.86
6.51	N282A	8.76±0.08	73.85±4.13*	83.74±10.51	N280A	6.53±0.36	82.13±9.85	149.76±6.71
6.52	H283A	7.14±0.14***	78.53±4.91*	48.61±6.28	H281A	6.02±0.22	32.65±9.35*	211.66±12.67
6.55	Y286A	6.42±0.07***	116.03±7.01	47.01±3.35	Y284A	5.46±0.25	79.50±1.73**	NT
6.58	R289A	NA	NA	4.81±4.97	R287A	NA	NA	130.96±24.39
6.58	R289H	6.30±0.13***	82.99±2.61*	98.05±6.46	R287H	5.27±0.09***	47.19±25.23	43.88±17.99
6.61	N292	NT	NT	81.52±6.00	H290A	6.05±0.15	144.20±27.65	269.90±18.37
ECL3	Y293A	8.15±0.12	90.39±15.12	122.45±4.60	Y291A	5.72±0.21	101.72±3.61	260.51±74.72
ECL3	D297A	7.80±0.20	109.32±16.48	24.16±0.70	D295A	5.49±0.13**	83.46±21.40	123.10±17.66
ECL3	P298A	8.21±0.15	88.68±10.42	42.75±6.08	T296A	6.40±0.27	121.95±44.58	275.41±15.80
7.31	H302A	7.37±0.13*	86.72±13.19	48.07±1.78	H300A	5.62±0.08***	124.90±23.70	145.88±30.42
7.32	M303A	8.65±0.03	107.57±13.70	19.15±1.44	F301A	5.71±0.25	150.32±50.02	77.74±7.57
7.39	R310A	6.69±0.15***	106.12±8.98	37.09±2.85	R308A	5.52±0.12**	10.90±10.26*	214.81±35.21
7.43	F314A	8.37±0.05	98.02±18.40	64.70±4.36	F312A	6.03±0.03***	74.81±15.01	85.97±10.67
-	NMB	6.21±0.06***	106.55±5.03	100±4.14	GRP(14-27)-NH_2	8.30±0.12***	103.10±1.54	100±29.335
